# Modern Methods of Antiseptic Wound Treatment

**Published:** 1891-12

**Authors:** 


MODERN METHODS OF ANTISEPTIC WOUND TREATMENT. Johnson & Johnson, New York.

In calling attention to this little book, printed for gratuitous distribution, we believe we are performing for our readers a genuine service, for the work is unquestionably valuable, containing so many hints from such authorities as Askew, Lister, J. William White, Deaver, Stephen Smith, N. Senn, McGuire, Morton, Stimon and others, relating to the practical application of antiseptics in wound treatment, that the surgeon must be more than well posted not to be able to find something of interest in its pages.

The book contains portraits of a large number of the world's leading surgeons, but is valuable chiefly on account of the full details concerning the application of antiseptics in ordinary wound dressing—such details as are not commonly found in surgical text-books.

Messrs. Johnson & Johnson are entitled to no little credit for the production of matter so useful, etc.

They will send a copy to any physician making application.

